# Supplementary material for: Changes in Sleep Quality, Sleep Duration, and Sickness Absence: A Longitudinal Study with Repeated Measures
Source: Healthcare (Basel). 2024 Jul 11;12(14):1393. doi: 10.3390/healthcare12141393 (PMC11275330; doi:10.3390/healthcare12141393)
Supplement: Supplementary file 1 [file healthcare-12-01393-s001.zip › healthcare-3082864-supplementary.pdf]

Supplementary Table S1. Mean ±SD individuals across measurements.

	**Average across time: mean±SD across individuals Total group** N=5377	**Average across time: mean±SD across individuals Males** N=2437	**Average across time: mean±SD across individuals Females** N=2940
**Work demands (1-4 high)**	0.34±0.20	0.33±0.20	0.34±0.21[a]
**Lack of work control (1-4 low)**	0.23±0.15	0.23±0.15	0.23±0.15
**Physical workload (1-6 high)**	0.44±0.50	0.45±0.50	0.43±0.49
**Poor sleep quality (1-6 poor)**	0.52±0.36	0.48±0.33	0.54±0.37[c]
**Sleep duration: work (hrs)**	0.45±0.42	0.46±0.43	0.45±0.40
**Sleep duration: days off (hrs)**	0.57±0.42	0.59±0.45	0.56±0.40
**Bedtime, work**	0.40±0.70	0.40±0.68	0.40±0.71
**Time of rising, work**	0.44±0.87	0.45±0.97	0.42±0.78
**Bedtime, days off**	0.40±0.70	0.40±0.68	0.40±0.71
**Time of rising, days off**	0.48±0.42	0.49±0.44	0.48±0.39
